# One-dimensional diffusion of TrpR along DNA enhances its affinity for the operator by chemical ratchet mechanism

**DOI:** 10.1038/s41598-021-83156-6

**Published:** 2021-02-19

**Authors:** Takashi Kinebuchi, Nobuo Shimamoto

**Affiliations:** 1 National Institute of Genetics, Mishima, Shizuoka 411-8540 Japan; 2 Department of Genetics, School of Life Science, The Graduate University for Advanced Studies, Mishima, Shizuoka 411-8540 Japan; 3 Present Address: Olympus Corporation, Quality Assurance and Regulatory Affairs, 2951 Ishikawa-machi, Hachioji-shi, Tokyo 192-8507 Japan; 4 Veritas Kitayama, 30-1-104 Shimogamo-Minamishiba-cho, Sakyoku Kyoto, 606-0841 Japan

**Keywords:** Biochemistry, Biophysics, Molecular biology

## Abstract

Several DNA-binding proteins show the affinities for their specific DNA sites that positively depend on the length of DNA harboring the sites, i. e. antenna effect. DNA looping can cause the effect for proteins with two or more DNA binding sites, i. e. the looping mechanism. One-dimensional diffusion also has been suggested to cause the effect for proteins with single DNA sites, the diffusion mechanism, which could violate detailed balance. We addressed which mechanism is possible for *E. coli* TrpR showing 10^4^-fold antenna effect with a single DNA binding site. When a *trpO*-harboring DNA fragment was connected to a nonspecific DNA with biotin-avidin connection, the otherwise sevenfold antenna effect disappeared. This result denies the looping mechanism with an unknown second DNA binding site. The 3.5-fold repression by TrpR in vivo disappeared when a tight LexA binding site was introduced at various sites near the *trpO*, suggesting that the binding of LexA blocks one-dimensional diffusion causing the antenna effect. These results are consistent with the chemical ratchet recently proposed for TrpR-*trpO* binding to solve the deviation from detailed balance, and evidence that the antenna effect due to one-dimensional diffusion exists in cells.

## Introduction

In the chromosomes, there are a limited number of DNA sites where a protein binds with high affinities for its complex to perform the genetic commands, specific sites. These proteins are also known to bind to most parts of DNA, nonspecific sites, in much weaker affinities. The complexes with these sites are generically called specific and nonspecific complexes. In this view, DNA can be considered as a sequence of specific and nonspecific sites with nonspecific sites occupying most parts of DNA. If the high affinity for a specific site is determined solely by its local sequence or structure, nonspecific sites should have no function other than competing against specific sites for such a protein.

Contrary to this simple competitive view, the affinities of several proteins are reported to increase when their specific sites exist on longer DNA. This length effect was compiled under the name of “antenna effect” irrespective of the mechanisms^1^. The earliest report was for LacI and the enhancement of the affinity was 10–1,000 fold^2,3^. Since a LacI molecule is homotetrameric and can bind to two specific sites on DNA simultaneously, the change in the apparent affinity has been attributed to the additional stabilization by the second intramolecular binding to later found operators on the same DNA molecule^4^. The longer the DNA, the more frequent formation of the intramolecular DNA loop stabilizing the complex. Thus, this looping mechanism provides a clear explanation of the antenna effect for a protein molecule with two or more DNA-binding sites like LacI^5–9^. In contrast, the application of the mechanism for the proteins with single DNA-binding sites is difficult. In the case of the homodimeric bacterial repressor, only a single binding site is formed from two helix-turn-helix motifs of two subunits. Thus, the putative second DNA-binding site must be attributed to an unfound site or arbitrary surface of the protein, but such sites have not so far became evidenced. Moreover, there are accumulating examples in structural biology where a significant stabilization is attributed to only specific molecular interactions but not ones with an arbitrary surface. Thus, the looping mechanism for a protein with a single DNA-binding site is still questioned.

The antenna effect for a protein with single binding site, *E. coli Eco*RI methyltransferase, was found by Surby and Reich^10^ as the first systematic study of the effect. By using gel-shift assay, they showed that the affinity for whole DNA fragments increased 20-fold as the length increased from 14 to 775 bp, while the observed dissociation rate constant was independent of the length. From the results, they proposed a mechanism based on the accelerated association by one-dimensional diffusion along DNA with a declared reservation of the “violation of the thermodynamic rule”, without further comment.

This rule is usually called detailed balance and prohibits the existence of net circulation flow among reaction components at equilibrium, and thus claims that acceleration of a rate must be accompanied by the acceleration of its reverse rate of every step in the equilibrated reaction, maintaining the binding affinity for the specific site unchanged. This rule has been strictly established in the timescale at equilibrium in statistical mechanics^11^. Since the affinity is also determined at equilibrium, the rule seemingly denies the antenna effect caused by one-dimensional diffusion.

We found that the “equilibrium” required for detailed balance is different than the one required for determining an affinity. The former is stricter and must be established on the level of molecular systems. In contrast, the latter is a macroscopic steady state of the ensemble average of the molecular systems and does not necessarily demand the establishment of the former. As discussed later in more detail, if some of the reaction component molecules alter their intrinsic affinity/stability according to oscillating conformational changes with random phases, the latter is possible to be established to show a macroscopic affinity but the rule does not hold in the non-equilibrium state. We named the mechanism “chemical ratchet”, which is consistent with the 10,000-fold antenna effect of TrpR-*trpO* binding^12^*.*

Since there is neither evidence for nor counterevidence against the looping mechanism for TrpR-*trpO* binding, we designed a connection of DNA segments with avidin–biotin, which allows DNA looping but hampers one-dimensional diffusion by disrupting B-DNA helical structure. When a *trpO*-harboring DNA segment was connected to a nonspecific DNA segment with the intact phosphodiester bonds, the antenna effect was seven fold. In contrast, when they are connected with avidin–biotin, no antenna effect was observed (one-fold). We thus concluded that one-dimensional diffusion, but not DNA looping, is the cause of the length dependence of the affinity of TrpR for *trpO*.

The existence of one-dimensional diffusion in cells has been suggested for *Eco*RV by the observed correlation between the first-order cleaving rates in vitro and the titer values of bacteriophage lambda in vivo for the wild-type and mutant enzymes^13^. We examined the existence of antenna effect caused by one-dimensional diffusion in vivo by our block method. We inserted a LexA binding site with various affinities at various distances from the *trpO* regulating the expression of *lacZ* monitor. The measured expression of *lacZ* showed a good correlation to the LexA affinity for the sites, providing evidence for the existence of one-dimensional diffusion as well as antenna effect due to the diffusion in vivo, suggesting the cross-talk between two proteins at a distance on DNA.

## Results

### Elimination of DNA looping from the major mechanism of antenna effect

The dissociation equilibrium constant *K*_*d*_ of TrpR-*trpO binding* is defined as

1 $$ K_{d} \equiv \left( {affinity} \right)^{ - 1} \equiv \frac{{\left[ {{\text{free}}\,{\text{TrpR}}} \right]\left[ {{\text{free}}\,trpO} \right]}}{{\left[ {{\text{TrpR}} - trpO\,{\text{complex}}} \right] }}\quad {\text{at}}\,{\text{equilibrium}}. $$

All the symbols and their definitions are listed in Table S1 (Supplementary). Since the hydroxyl radical footprinting^14^ can directly quantify the amount of TrpR protein complexed at *trpO* site, we first determined the ratio of [TrpR-*trpO* complex] to [*trpO*]_total_ at equilibrium at various [*trpO*]_total_ and then determined the *K*_*d*_ value according to Eq. (2) by the least square fit as described in our preceding paper^12^. In our experimental condition the amount of TrpR was in excess over *trpO*, and thus [free TrpR] was approximated by [TrpR]_total_.

2 $$ \left[ {{\text{TrpR}} - trpO\,{\text{complex}}} \right] = \frac{{\left[ {{\text{free}}\,{\text{TrpR}}} \right]\left[ {trpO} \right]_{total} }}{{K_{d} + \left[ {{\text{free}}\,{\text{TrpR}}} \right]}} . $$

When the 36 bp DNA harboring *trpO* at its center is connected to 232 bp nonspecific DNA fragment, the affinity is enhanced, and 7.4-fold antenna effect was observed (Table 1). We tested the looping mechanism by connecting the two fragments with biotin-avidin binding. Since this connection preserves DNA looping, the antenna effect by the looping mechanism should be preserved. The looping may even enhance the effect because tetrameric avidin molecule can connect the two DNA fragments at an angle much smaller than 180°. Moreover, the flexible (–CH_2_–)_9_ residue in the biotin linker may facilitate DNA looping. In fact, a similar avidin connection proved the enhancer action mediated by DNA looping^4,15^. In contrast, this connection is expected to hinder one-dimensional diffusion of a protein along DNA at the joint, because of the diameter of avidin being much larger than that of DNA and because of the positively charged avidin surface opposite to the DNA surface. The sliding, a mode of one-dimensional diffusion in which a protein molecule tracks the DNA groove, especially, should be blocked by the disruption of the DNA grooves.

The obtained results clearly showed that the 7.4-fold antenna effect is caused by one-dimensional diffusion but not by DNA looping: the disappearance of the effect by biotin-avidin connection (Table 1). Moreover, we confirmed that the addition of biotin-avidin at the end of 36 bp *trpO* DNA did not change its affinity for TrpR (Table 1).

### Effect of the binding of LexA protein near the operator in vivo

It is not easy to get evidence for the existence of one-dimensional diffusion in vivo. The existence of antenna effect in vivo may be more difficult. In vitro, we have hindered one-dimensional diffusion by introducing avidin between two DNA fragments as shown above. We then used in vivo a similar method to hinder one-dimensional diffusion by introducing LexA protein and LexA binding sites near *trpO* in a low copy plasmid DNA. If the antenna effect exists also in vivo, the formation of the tight LexA-DNA complex near *trpO* is expected to decrease the affinity of TrpR for *trpO*, and then suppresses the repression by TrpR. Therefore, disappearance of the antenna effect due to blocked one-dimensional diffusion, similar to the in vitro experiment described above, can be detected. The block should consequently decrease the affinity of TrpR for *trpO*, enhancing the expression of the monitor gene *lacZ* cloned downstream of *trpO*. In this way, we examine the existence of one-dimensional diffusion as well as the antenna effect due to the diffusion.

**Figure 1:**
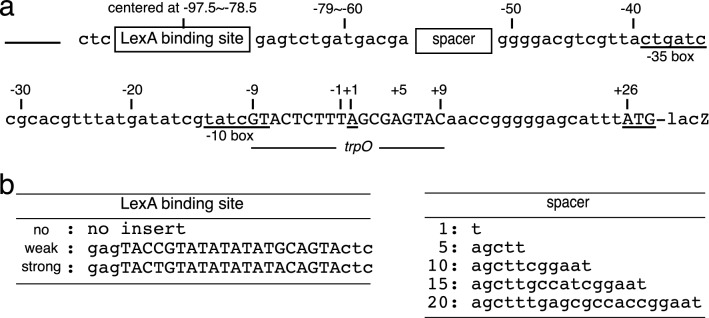
Structures of the *trpO* site with the LexA binding sites upstream (a). The position of a LexA binding site inserted and the position of a spacer are indicated with the *trpO* ordinate, and the functional elements are indicated as “-35 box”, “-10 box”, and “*trpO*”. Transcription starts at + 1. (b). The sequences of the LexA binding sites and the spacers shown in Panel A and Table 2.

A series of low-copy mini-F plasmids^16^ harboring a LexA binding site^17^ was constructed. The part upstream from the intact *trpO*-*trpR* promoter was remain intact and was followed by a *lacZ* reporter gene (Fig. 1). The bound LexA is expected to block the sliding of TrpR into the *trpO* to diminish the antenna effect, activating *lacZ* transcription from the *trpO*-*trpR* promoter. But a similar block also could be induced by direct contact between the bound LexA and/or the initiating RNA polymerase and/or TrpR. Such protein–protein contacts are known to require the presence of the protein molecules on the same face of the double-stranded DNA as shown for λ repressor cI^6^ and RNA polymerase^7,18^, resulting in an iterative pattern of the presence and the absence of the effect at every 5–6 bp, a half pitch of DNA helix. Therefore, the spacing was changed between the LexA site and the promoter among the 5 constructs (Fig. 1).

**Table 1:**
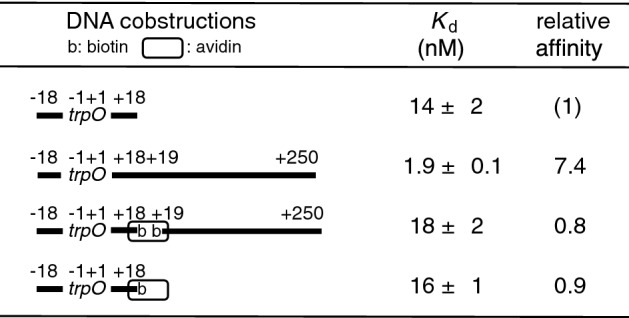
Loss of the antenna effect by biotin-avidin connection.

The *trpO* of *trpR* gene locates at the center of 36 bp DNA. It is connected with the native phosphodiester bond or with biotin-avidin–biotin. The base sequence from − 18 to + 250 is genomic.

The control strain harboring the plasmid with no LexA site showed a repression by TrpR of about fourfold (data lines 1 and 2 in Table 2) as previously reported on a λ lysogen harboring the *trpO*^19^, whereas the strains harboring plasmids carrying the consensus LexA site showed the expected disappearance of repression irrespective of the spacings (lines 3–7). The independence of the iterative pattern at every 5 bp (lines 3–7) indicated that the loss of repression was not due to the direct contact of the bound LexA to RNA polymerase or TrpR^7^. Furthermore, a weakened LexA target allowed intermediate repression (lines 8 and 9), and overproduction of TrpR recovered the repression depending on the affinities of LexA site (lines 10–13). These results indicate that the tight binding of LexA decreased the affinity of TrpR for the *trpO* at a distance. The recovery of repression at a higher level of TrpR in vivo is consistent with the mechanism that the affinity was decreased enough by the partial blocking of sliding only from upstream. These results are consistent with the model that the one-dimensional diffusion exerts antenna effect.

**Table 2:** Repression of lacZ expression by TrpR and the effect of LexA binding upstream of *trpO.*

LexA binding site	Position (center)	Spacer (bp)	LacZ activity^a^		Repression (fold)
			+Trp	−Trp	
Null	− 78.5	1	62 ± 2	262 ± 11	4.2
Null	− 82.5	1	77 ± 2	278 ± 11	3.5
Consensus	− 78.5	1	255 ± 4	260 ± 8	1.0
Consensus	− 82.5	5	272 ± 2	267 ± 5	1.0
Consensus	− 87.5	10	262 ± 8	250 ± 9	1.0
Consensus	− 92.5	15	266 ± 10	272 ± 9	1.0
Consensus	− 97.5	20	243 ± 4	272 ± 8	1.1
Weak	− 78.5	1	145 ± 4	255 ± 5	1.8
Weak	− 82.5	5	165 ± 3	266 ± 3	1.7
Null^b^	− 82.5	5	12 ± 5	230 ± 5	19
Consensus^b^	− 82.5	5	111 ± 4	229 ± 6	2.1
Weak^b^	− 82.5	5	40 ± 5	231 ± 2	5.8

^a^Averaged Miller unit in triplicated measurements.

^b^With a multicopy plasmid overproducing TrpR, that harbors the pBR322 origin.

## Discussion

The DNA looping mechanism has a characteristic dependence on DNA length. It becomes difficult for the DNA length shorter than the persistent length, ca. 50 nm or 150 bp, since a looping of shorter DNA costs energy because of rigidity of double-stranded DNA^20–22^. This length limitation may be mitigated in two cases. In the first case, a flexible peptide links two DNA-binding sites as in the case of *E. coli* AraC^5^. In the second, relevant energy is supplied by two specific interactions in the case of the *λ* repressor cI^6^ and *E. coli* LacI^4^ or by a specific binding of another protein in the case of *E. coli gal* repressor^9^. However, TrpR molecule, 2–3 nm for a homodimer, is too small and has neither a flexible peptide linker nor extra binding surfaces generating the relevant energy on the tested DNA fragments. Moreover, when the specific site is cloned at the center of DNA as in our experiments, looping DNA should be facilitated for DNA longer than twice the persistent length, 300 bp. However, the major increase in the affinity of TrpR, 720 fold, was observed as increasing DNA length from 18 to 200 bp^12^, for which DNA looping is difficult to occur. Thus, consideration of DNA rigidity contradicts the looping mechanism of TrpR.

Since the one-dimensional diffusion and the looping mechanisms predict different dependences on DNA length of association and dissociation rates, we tried to measure their dependences. However, the TrpR-*trpO* binding cannot be fluorescently monitored^23^, and the rates were too fast to be determined with other available methods.

**Figure 2:**
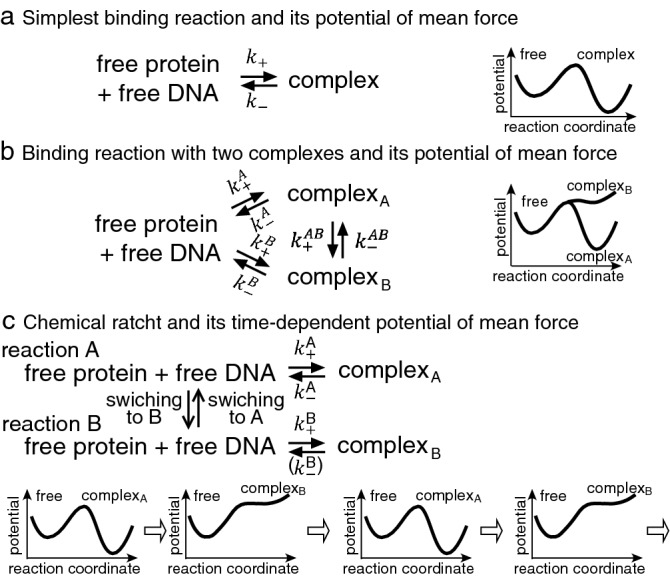
Comparison of chemical ratchet mechanism with a conventional description of a binding reaction. The binding reaction composed of two reactants and a product complex (free DNA, free protein, and the complex) is here considered. (**a**) The simplest binding reaction and its potential of mean force along the reaction coordinate in conventional description. (**b**) The product complex exists in two forms, the more stable complex_A_ and a less stable complex_B_ in conventional description. (**c**) Chemical ratchet in which two or more potentials of mean force alternate. During reaction A, the association is dominant to form more stable complex_A_ which is more stable, while during reaction B, the dissociation becomes dominant to dissociate unstable complex_B_, generating an alternating reaction flow. In the dissociation reaction of reaction B, a rate constant, $k_{ - }^{B}$, may not be able to be defined because the complex_B_ may not in the local minimum of the potential.

The largest difficulty of the diffusion mechanism as a cause of antenna effect is its deviation from detailed balance. This rule holds in the timescale where all the degrees of freedom of the reaction molecules become stationary^11^, conventionally expressed as “at equilibrium”, which might cause misunderstanding if the concept of timescale is not taken into account^24^. We here defined the timescale of a reaction, the summed average times cost in its forward and reverse steps. If a reactant is in excess over the other, the timescale of the binding can be defined as $\left\{ {k_{ + } \left[ {{\text{excess}}\,{\text{reactant}}} \right] + k_{ - } } \right\}^{ - 1}$, which is the inverse of decay time of the binding reaction shown in Fig. 2a, indicating how fast the reaction reaches its stationary state.

In Fig. 2, we schematically show three mechanisms of bimolecular binding. Among them, the simplest basic protein-DNA binding reaction is shown in Panel (a), while the reaction with additional forms of complex is shown in Panel (b). The potential mean force along the reaction coordinate shows local minima of the number of states of free reactants and complexes and the potential is time independent. In panels (a) and (b), the slowest timescale is that of the binding reactions and others of internal degrees of freedom are faster. Therefore, detailed balance holds on the slowest timescale^14^, binding in Panels (a) and (b).

In chemical ratchet, the potential of mean force changes in time: reaction A and reaction B alternate with more stable complex_A_ and with less stable conplex_B,_ respectively, in the example shown in Panel (c). Although the schematic illustrations in Panels (b) and (c) may look similar, there is a critical difference. Two complexes are converted to each other via the direct $k_{ \pm }^{AB}$ pathway and/or the stepwise $k_{ \pm }^{A}$ and $k_{ \pm }^{B}$ pathways via the free state in Panel (b). The conversion is described with rate equations. In contrast, in Panel (c), chemical ratchet, two complexes are alternative. Complex_A_ cannot exist in reaction B, and complex_B_ cannot exist in reaction A, making the switching unable to be described with a single set of rate equations where all steps are stochastic. During reaction A, the equilibrium is inclined toward complex_A_, while during reaction B, it is inclined toward the free state because of unstable complex_B_. Therefore, in a microscopic view, the dominant reaction during reaction A is association, while that during reaction B is dissociation, making alternating reaction flow.

The phases of switching are random for each DNA molecule and, and thus the ensemble average essentially becomes time independent due to the cancellation among the microscopic differences, resulting in a stationary state at non-equilibrium. Since detailed balance requires perfect equilibrated stationary state, this non-equilibrium state of chemical ratchet is indifferent to the detailed balance of the binding reaction.

We are now ready to explain the length dependence of *K*_*d*_ in Eq. (1), the antenna effect of TrpR. One-dimensional diffusion of TrpR along DNA can accelerate its association to *trpO* site as well as its dissociation from the site. If reaction A is more dependent on one-dimensional diffusion, while reaction B is less, the association is more accelerated for longer DNA, while dissociation not as much. Therefore, the longer the DNA, the smaller the value of *K*_*d*_.

**Figure 3:**
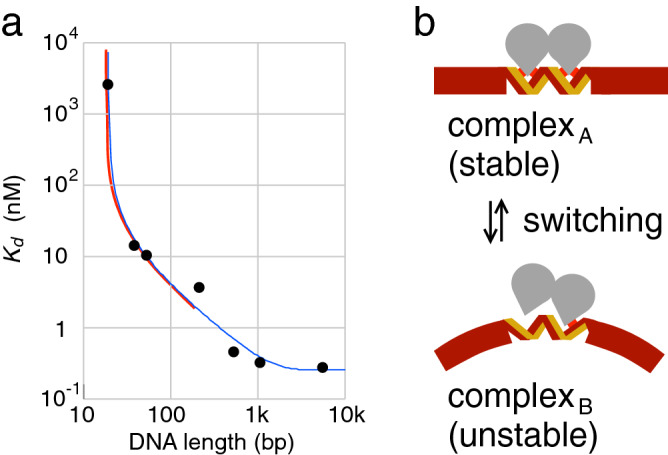
(**a**) The theoretical length dependence (red line) with the parameters fitted to the experimental results agreed with that obtained from the stationary solution of the reaction–diffusion differential equation (blue line). The experimentally determined values of *K*_d_ (filled circles) and the theoretical curve in blue were taken from Ref^12^. The size of the *trpO* site is a common parameter and showed essentially the same value in the two analyses to be 18 bp. (**b**) One of the possible molecular models for the chemical ratchet of TrpR (gray homodimer) binding to *trpO*. DNA is illustrated as a thick brown bar and *trpO* site is illustrated as double stranded DNA to emphasize the specific interaction (red box) in the major DNA groove. ComplexA is stable, while complexB is unstable due to the specific interaction damaged by an infrequent DNA bending at *trpO* (see the text).

The length dependence of *K*_d_ can be kinetically derived under the condition that DNA length is short enough for one-dimensional diffusion to be equilibrated. Furthermore, we also assume that the isomerization between the specific and nonspecific complexes is also equilibrated in the timescale of the binding. The kinetic changes of the complex and free components are calculated and averaged over a cycle of the switching to obtain *K*_d_ (Supplementary). The calculated length dependence well agreed with the theoretical curve obtained as a stationary solution of a stricter differential equation ^12^. As shown in the red line in Fig. 3a, the calculation is only significant for DNA length shorter than the sliding distance (ca. 600 bp) previously determined^12^ because of the assumption of rapid diffusion.^.^

To support the reality of the chemical ratchet model, we should propose at least one possible molecular model based on the knowledge on protein-DNA binding (Fig. 3b). Complex_A_ and complex_B_ have straight and bent DNAs at *trpO*, respectively. Complex_A_ is a stable complex, while complex_B_ is an unstable intermediate. Complex_B_ tends to dissociate in concerted manner with the bending because DNA bending at *trpO* in the complex distorts the DNA grooves and disrupts the specific interactions between TrpR and *trpO* DNA. The reactions from complex_B_, its dissociation or straightening DNA, cannot be described with rate equations because complex_B_ is not at a significant local minimum on the potential of mean force. The DNA bending as well as straightening in the complex are expected to occur much less frequently than those of naked DNA due to the specific protein-DNA interactions, providing the degree of freedom slower than that of binding. Therefore, bending DNA and straightening DNA can be the switching of chemical ratchet. There are, however, a lot of other possible models for a chemical ratchet.

The research on TrpR and *trpO* has a long successful history^25^ but there remains a problem on the specificity and the level of TrpR in cells. In *E. coli* cells, TrpR must significantly saturate the *trpO* site to show its function, and thus the intercellular concentration of TrpR must be close to values of *K*_*d*_. If we suppose the concentration is equal to *K*_*d*_, and suppose the affinity for nonspecific site is S-fold weaker than that of the *trpO*, the amount of the specific complex at *trpO* per genome and that of the complex at a nonspecific site are respectively,

3 $$ \frac{{\left[ {TrpR} \right]}}{{ K_{d} + \left[ {{\text{TrpR}}} \right]}} = \frac{1}{2}\,{\text{and}}\,\frac{{\left[ {TrpR} \right]}}{{ SK_{d} + \left[ {{\text{TrpR}}} \right]}} = \frac{2}{S + 1}. $$

When N is the number of exposed nonspecific sites, there should be at least $\frac{1}{2} + \frac{2N}{{S + 1}} \sim \frac{2N}{S}$ molecules of TrpR per genome. The value of N is at least 10^6^, because about a half of the DNA in *E.coli* cells is shown to be exposed by the quantitative footprinting assay of IHF in vivo^26^. In the gel-retardation assay using 90 bp *trpO* DNA fragment in vitro, the value of *S* has been determined to be ca. 300 because half of DNA molecules retain one nonspecific complex per specific complex at [*TrpR*] = 300 *K*_*d*_^23^. From the values of N and S, there should be at least 3,000 TrpR molecules per genome existing in cells.

However, this estimate is contradictory to the measured level of TrpR, ca. 300 molecules per genome^27^, by an order smaller than those required to occupy *trpO*. The antenna effect of TrpR can solve this problem, if the sliding distance in vivo is 500 bp or longer, because the value of *S* in the cell is expected to be an order of magnitude larger than that for 90 bp (Fig. 3a).

Antenna effect, which can be driven by chemical ratchet, may provide a novel interpretation to experimental observation of DNA sequence dependency of protein binding affinity. Lukatsky and his colleague found that several proteins enhance the affinity of the specific complex depending on the sequence of DNA segments flanking the specific site in vivo and in vitro^28–31^. The enhancing sequences were repetitive or homopolymeric sequence, which had been known to facilitate one-dimensional diffusion^32^, as the authors estimated the contribution of the diffusion.

The studies on one-dimensional diffusion have been developed with the focus on best combination of different diffusion modes^33^, the overlooking of the specific site^34^, speed-selectivity paradox^35,36^, and so on. Since the acceleration is a kinetic effect, the contribution of the acceleration is temporally limited to the phenomena with timescales similar to the accelerated association rates, say seconds or less, as long as detailed balance holds. Chemical ratchet can link the rapid kinetic effect to the physiological phenomena with much slower timescales through antenna effect. In this way, one-dimensional diffusion may contribute to gene expression and its regulation in more general way than the kinetic effect.

## Materials and methods

### Protein and DNA

*E. coli* TrpR protein was provided by Dr. Jeannette Carey. The 36 bp and 268 bp DNAs were prepared with PCR and purified by electrophoresis in an 8% polyacrylamide gel, followed by simple diffusion from the crushed gel slices. The biotin-attached 36 bp and 232 bp DNA fragments were prepared using 5′-end biotinylated primers. The 36 bp biotinylated DNA was next preincubated with a two-fold excess of avidin (avidin DN from Upstate Biotechnology) for 1 h, then mixed with a two-fold excess of 232 bp biotinylated DNA. Avidin from some other commercial sources could not be used because of nonspecific binding of DNA. The 36 + 232 bp DNA connected by biotin-avidin was isolated by using 8% polyacrylamide gel.

A series of DNA fragments harboring a LexA binding site were prepared by PCR using different primers and inserted at a *Xho*I site of pFF6 plasmid, which carries *lacZ* with dual origins of pBR101 and the miniF^18^. Single-copy plasmids were then prepared by replacing the pBR origin with a *trpO* fragment at the *Hin*d III site of the plasmid. The plasmids were used to transform *E. coli* MC4100 strain and the transformants were grown in M9 medium with or without 0.25 mM L-tryptophan. Growth was rapidly halted at OD_600_ = 0.5–0.7 by immersing an aliquot in liquid nitrogen. The samples were then kept frozen until the standard β-galactosidase assay was performed.

### Hydroxyl radical footprinting

All the binding and footprinting experiments were performed as already described^15^. The Fenton reagent was freshly prepared from concentrated solutions and all the measurements for a DNA were finished before the aging period that had been determined in preliminary experiments. Reaction was stopped by an addition of glycerol to 20%. To satisfy the single-cutting condition, the period of cleavage reaction was limited so that less than 20% of the full-length DNA fragment had disappeared according to the results of preliminary experiments. Fitting the data to Eqs. (1) and (2) was carried out by the least-squares method with MacCurveFit 1.5 and the standard deviations were obtained from the sum of the squared errors.

### Measurements of TrpR-*trpO* binding in vivo

In the measurement in vivo, determination of the Mirror unit, required many cautions. The observed value was dependent on the lot of culture media and the recovery procedure of *E. coli* cells from its stock solution. Thus we prepared a large volume of the media and kept using the same lot. The cells were recovered three times in the fresh lot with the same dilutions into the fresh medium and the same shaking process taking three days. Growth was rapidly halted at OD_600_ = 0.5–0.7 by immersing an aliquot in liquid nitrogen. The samples were then kept frozen until the standard β-galactosidase assay was performed. This whole process was repeated three times as listed in Table 2.

## Supplementary Information

Supplementary Information.
