# Supplementary material for: One-dimensional diffusion of TrpR along DNA enhances its affinity for the operator by chemical ratchet mechanism
Source: Sci Rep. 2021 Feb 19;11:4255. doi: 10.1038/s41598-021-83156-6 (PMC7896080; doi:10.1038/s41598-021-83156-6)
Supplement: Supplementary file 1 — Supplementary Information. [file 41598_2021_83156_MOESM1_ESM.docx]

**Supplementary Information for**

**“One-Dimensional Diffusion of TrpR along DNA Enhances Its Affinity for The Operator by Chemical Ratchet Mechanism”**

**Takashi Kinebuchi^1^ and Nobuo Shimamoto*^2^**

National Institute of Genetics, and Department of Genetics, School of Life Science, The Graduate University for Advanced Studies, Mishima, Shizuoka 411-8540, Japan

^1^ Present address: Olympus Corporation, Quality Assurance and Regulatory Affairs, 2951

Ishikawa-machi, Hachioji-shi, Tokyo 192-8507, Japan

^2^Present address: Veritas Kitayama, 30-1-104 Shimogamo-Minamishiba-cho, Sakyoku, Kyoto, 606 0841 Japan

1. Table S1**.** Symbols in the main text

| symbol | definition | appearance |
| --- | --- | --- |
| $K_{d}$ | dissociation equilibrium constant of TrpR-*trpO* binding: (affinity)^-1^ | Eq. 1 |
| $\left[ free TrpR \right]$ | concentration of free TrpR (saturated with the corepressor L- tryptpophan) at equilibrium | Eqs. 1,2 |
| $\left[ \mathrm{free} trpO \right]$ | concentration of empty *trpO* site at equilibrium | Eqs. 1,2 |
| [TrpR-*trpO*  complex] | concentration of TrpR-*trpO* complex at equilibrium | Eqs. 1,2 |
| $k_{+}$ | association rate constant of general binding reaction | Fig. 2a |
| $k_{-}$ | dissociation rate constant of general binding reaction | Fig. 2a |
| $k_{+}^{A}$ | association rate constant to produce complex_A_ of general binding reaction (2b) or reaction A in chemical ratchet (2c) | Fig. 2b,c |
| $k_{-}^{A}$ | dissociation rate constant to produce complex_A_ of general binding reaction (2b) or reaction A in chemical ratchet (2c) | Fig. 2b,c |
| $k_{+}^{B}$ | association rate constant to produce complex_B_ of general binding reaction (2b) or reaction A in chemical ratchet (2c) | Fig. 2b,c |
| $k_{-}^{B}$ | dissociation rate constant to produce complex_B_ of general binding reaction (2b) or reaction A in chemical ratchet (2c) | Fig. 2b,c |
| N | number of vacant nonspecific sites per genome | near Eq. 3 |
| S | (affinity for *trpO*) / (affinity for a nonspecific site) | Eq.３ |

2. **Table S2. Symbols used in this supplementary**

| Symbol/term | definition | apperance |
| --- | --- | --- |
| Reaction A | one of switching mechanisms of chemical ratchet that involves both direct binding and indirect binding mediated one-dimensional diffushion producing stable TrpR*-trpO* complex, complex_A_. | Fig. S1b |
| Reaction B | one of switching mechanisms of chemical ratchet that involves only direct binding producing unstable TrpR*-trpO* complex, complex_B_. | Fig. S1c |
| complex_A_ | stable conformation TrpR*-trpO* complex in reaction A | Fig. S1 |
| complex_B_ | unstable conformation of TrpR*-trpO* complex in reaction B or TrpR*-trpO* complex in unstable transient configulation in reaction B | Eqs. 1,2 |
| *l* | number of nonspecific sites between *trpO* and an DNA end. *trpO* locates at the center of DNA | Eqs. 1,2 |
| *D* | diffusion constant of one-dimensional diffusion in the reaction A | Eqs. 1,2 |
| a | association rate constant$\times\left[ free TrpR \right]$ to a DNA site | Fig. 2a |
| d | dissociation rate constant of a nonspecific complex | Fig. 2a |
|  |  | Fig. 2a |
| e | isomerization rate constant to produce complex_A_ at *trpO* in reaction A | Fig. 2a |
| *d* | dissociation rate constant of a nonspecific complex in the reaction A | Fig. 2a |
| *d** | dissociation rate constant of complex_B_ in the reaction B | Fig. 2b,c |
| *e* | isomerization rate constant to produce complex_A_ at *trpO* in reaction A | Fig. 2b,c |
| *f* | isomerization rate constant to produce the nonspecific complex at *trpO* from complex_A_ in reaction A | Fig. 2b,c |
| *C_A_^0^* | concentration of complex_A_ at t=0 in reaction A | Fig. 2b,c |
| *C_B_^0^* | concentration of complex_B_ at t=T_1_ in reaction B | Eq.３ |
| T_1_ | time of switching reaction mechanism from reaction A to reaction B and thus reaction A lasts from t=0 to t=T_1_ | Fig. S2 |
| T_2_ | time of switching reaction mechanism from reaction B to reaction A and thus reaction A lasts from t=0 to t=T_1_ | Fig. S2 |
| *K*_d_ | dissociation equilibrium constant of TrpR-*trpO* binding: (affinity)^-1^ | Eq. S7 |
| m1 | offset parameter involved in logK_d_ (DNA length) | Eq. S8 |
| m2 | parameter involved in logK_d_ (DNA length) that means the site size of *trpO.* | Eq. S8 |

**3. Derivation of the dissociation equilibrium constant by the dynamic formulation of chemical ratchet under the assumption of rapid one-dimensional diffusion.**

In our previous paper^1^, we formulated a general set of equations composed of a diffusion equation and rate equations with DNA as a one-dimensional continuum. Since the equation is difficult to be solved analytically in time-dependent manner, we solved it in a stationary state and obtained the dependence of the dissociation constant of TrpR-*trpO* binding on DNA length. To discuss the dynamic properties of chemical ratchet, we must trade off between a dynamic solution and further limitations. We here supposed that one-dimensional diffusion is equilibrated in the timescale of the binding reaction. This happens when the DNA length is much shorter than the sliding distance.

The specific site, *trpO*, is sandwiched between two segments composed of $l$ nonspecific sites. Reaction A in the chemical ratchet involves one-dimensional diffusion (Fig. S1a). All the parameters used are listed in Table S2. Note that the association rate constant, $a$, involves the concentration $\left[ free TrpR \right]$ to have the same dimension as the dissociation rate constant of a nonspecific complex, $d$ (Fig. S2a). Moreover, $\left[ free TrpR \right]\approx\left[ \mathrm{TrpR} \right]_{tot}$ holds because TrpR exist in excess over DNA in our experiments. The isomerization between nonspecific and specific complexes are supposed to occur only at *trpO* site with the rate constants $e$ and $f$. This mechanism satisfies detailed balance as shown in the dissociation rate constant of the specific complex.

**Fig. S1 Reactions A and B in the chemical ratchet** The DNA harbors the *trpO* of *trpR* gene at the center and it is flanked by $l$ nonspecific sites on both sides. The processes described with rate equations are denoted with solid arrows, and the one-dimensional diffusion process with broken arrows. There are $2l$ parallel pathways of association/dissociation between the free state and nonspecific sites. **a**. Reaction A involves one-dimensional diffusion and the binding to *trpO* with a high affinity. The dissociation rate constant of complex_A_ satisfies detailed balance if reaction A is equilibrated as an isolated reaction. **b.** If the protein-DNA binding is much slower than both of the isomerization and the one-dimensional diffusion, the specific and all the nonspecific complexes can be merged into “DNA complex” in the timescale the binding. The nonspecific complexes at nonspecific sites, the nonspecific complex at *trpO*, and the specific complex participate in the merged complex at the ratio indicated above “DNA complex”. **c**. The reaction B composed of direct binding to *trpO* with a low affinity due to large *d**, and complex_B_ is far less stable than complex_A._

We assumed that the binding reaction is much slower than any of the isomerization between specific and nonspecific complexes at *trpO* as well as one-dimensional diffusion along DNA. The nonspecific complex at every position and the specific complex share the same common time course, and thus they are generically named “DNA complex”. Thus, in the timescale of the binding, the concentration of the specific complex exponentially converges to its equilibrated value in reaction A. All the $2l$ nonspecific complexes has the same concentration as a consequence of the rapid diffusion, and the ratio of the concentration of the specific complex to that of nonspecific complex at *trpO* equals to $e/{f\gg2l}$ because the specific complex is the major complex and $2l$ must be smaller than the sliding distance due to the rapid isomerization. Therefore, the amount of “DNA complex” is approximated by that of specific complex, complex_A._ Since the chemical ratchet is the switching between reactions A and B, a cycle of chemical ratchet is shown in Fig. S2.

**Fig. S2 Dynamic feature of the chemical ratchet switching** between reaction A and reaction B. The switching from A to B occurs at t=T_1_, and then that from B to A occurs at t=T_1_+T_2._.

Notably, the reaction A becomes kinetically equivalent to the simple one shown in Fig. S1c, although kinetic parameters are very different except the common association rate , a. This merged reaction component is composed of $2l+1$ nonspecific complexes and the specific complex with the fractions indicated in Fig. S1b. At binding equilibrium, these flows become equal to give

$\left[ DNA complex \right]^{\infty}\approx\left[ \mathrm{complex}_{A} \right]^{\infty}=\frac{ea}{fd+ea}\left[ DNA \right]_{tot}$ (S1),
and the time course of the reaction A is given as,

$\left[ \mathrm{complex}_{A} \right]=\frac{ea}{fd+ea}\left[ DNA \right]_{tot}+\left\{ C_{A}^{0}-\frac{ea}{fd+ea}\left[ DNA \right]_{tot} \right\}e^{-\left( 2l+1 \right)\frac{ae+fd}{e}t}$ (S2)

, where $C_{0}^{A}$ is the initial value.

The second reaction of the chemical ratchet, reaction B, gives the time course of $\left[ \mathrm{complex}_{B} \right]$ similary to Eq. (S2) with a faster dissociation rate constant $d^{*} than association (d^{*}\gg a)$. Thus the time course of complex_B_ is

$\left[ \mathrm{complex}_{B} \right]=\frac{a}{d^{*}}\left[ DNA \right]_{tot}+\left\{ C_{B}^{0}-\frac{a}{d^{*}}\left[ DNA \right]_{tot} \right\}e^{-d^{*}\left( t-T_{1} \right)}$ (S3).

The time average of [TrpR-*trpO* complex] during the period, is given by

$\frac{1}{T_{1}+T_{2}}\int_{0}^{T_{1}} \left[ {complex}_{A} \right]dt+\frac{1}{T_{1}+T_{2}}\int_{T_{1}}^{T_{2}+T_{1}} \left[ {complex}_{B} \right]dt=\frac{1}{T_{1}+T_{2}}\int_{0}^{T_{1}} \{C_{A}^{\infty}$

$+\left( C_{A}^{0}-C_{A}^{\infty} \right)exp\left( \lambda_{A}t \right)\}dt+\frac{1}{T_{1}+T_{2}}\int_{T_{1}}^{T_{2}+T_{1}} \left\{ C_{B}^{\infty}+\left( C_{B}^{0}-C_{B}^{\infty} \right)exp\left[ \lambda_{B}\left( t-T_{1} \right) \right] \right\}dt$

$=\frac{T_{1}C_{A}^{\infty}{+T}_{2}C_{B}^{\infty}}{T_{1}+T_{2}}+\frac{C_{B}^{0}-C_{A}^{0}}{T_{1}+T_{2}}\left\{ \frac{1}{\left( 2l+1 \right)\left( a+\frac{fd}{e} \right)}-\frac{1}{d^{*}} \right\}$ (S4)

, where we tentatively use

$C_{A}^{\infty}=\frac{ea}{fd+ea}\left[ DNA \right]_{tot} \mathrm{and} C_{B}^{\infty}=\frac{a}{d^{*}}\left[ DNA \right]_{tot}$. (S5)

Since $T_{1}\gg T_{2}$ is derived from $d^{*}\gg a$, the terms containing $\frac{1}{d^{*}}$, $C_{B}^{\infty}$, and $T_{2}$ are eliminated from Eq. S4, giving

time average of [TrpR-*trpO* complex] $\approx C_{A}^{\infty}-\frac{C_{B}^{0}-C_{A}^{0}}{\left( 2l+1 \right)\left( a+\frac{f}{e}d \right)T_{1}}$ and

time average of [free DNA]${\approx\left[ DNA \right]}_{tot}-C_{A}^{\infty}+\frac{C_{B}^{0}-C_{A}^{0}}{\left( 2l+1 \right)\left( a+\frac{f}{e}d \right)T_{1}}$ (S6).

From Eq. S5, the experimental dissociation constant, Eq. (1) in the text, is expressed as

$K_{d}{=\left[ \mathrm{TrpR} \right]}_{tot}\frac{\frac{f}{e}d\left( 2l+1 \right)T_{1}+\frac{C_{B}^{0}-C_{A}^{0}}{\left[ DNA \right]_{tot}}}{a\left( 2l+1 \right)T_{1}-\frac{C_{B}^{0}-C_{A}^{0}}{\left[ DNA \right]_{tot}}}=\left[ \mathrm{TrpR} \right]_{tot}\frac{\frac{fd}{ea}\left( 2l+1 \right)+\frac{C_{B}^{0}-C_{A}^{0}}{{aT_{1}\left[ DNA \right]}_{tot}}}{\left( 2l+1 \right)-\frac{C_{B}^{0}-C_{A}^{0}}{a{T_{1}\left[ DNA \right]}_{tot}}}$ (S7)

The constant, $\frac{C_{B}^{0}-C_{A}^{0}}{{aT_{1}\left[ DNA \right]}_{tot}}$, determines the dependence of $K_{d}$ on DNA length. If it is negligibly small, then $K_{d}\approx{\frac{fd}{ea}\left[ \mathrm{TrpR} \right]}_{tot}$, which is the conventional dissociation constant obtained with detailed balance. If the constant is much larger than $\frac{fd}{ea}\left( 2l+1 \right)$, $K_{d}\approx\frac{\left[ \mathrm{TrpR} \right]_{tot}}{2l+1}$ and this is the length-dependent $K_{d}$ in chemical ratchet mechanism.

In conclusion, if $T_{1}\gg T_{2}$ and the dimension-less constant $\frac{C_{B}^{0}-C_{A}^{0}}{{aT_{1}\left[ DNA \right]}_{tot}}\gg\frac{fd}{ea}\left( 2l+1 \right)$,

${\log K}_{d}\approx m1-\log\left( 2l+1 \right)=m1-\log\left\{ \left( DNA length+1-site size of trpO \right) \right\}$

$\approx m1-\log\left\{ 1+\left( DNA length-m2 \right) \right\}$ (S8),

where $1=log\frac{\left[ \mathrm{TrpR} \right]_{tot}}{a}\frac{C_{B}^{0}-C_{A}^{0}}{{aT_{1}\left[ DNA \right]}_{tot}}, m2=\frac{C_{B}^{0}-C_{A}^{0}}{a{T_{1}\left[ DNA \right]}_{tot}}$ . The length of nonspecific DNA $l$ satisfies $2l=\left( DNA length \right)-\left( site size of trpO \right)=\left( DNA length \right)-m2$. This is the case the chemical ratchet introduces the dependence on DNA length. It looks odd for m1 to include an experimental parameter $\left[ \mathrm{TrpR} \right]_{tot}$, but the ratio, $\frac{\left[ \mathrm{TrpR} \right]_{tot}}{a}$ , is the inverse of the secondary association constant from bulk which contains no experimental parameters. Eq. (S8) are best fit to the experimental values of $K_{d}$with m1=2.58 ± 0.01 and m2=17.8 ± 0.1 (bp) by using KaleidaGraph (Synergy Software)(Fig. 3a). The latter is the site size of *trpO* and also one of the four fitting parameters used in the stationary-state solution in the reference 15 in the text) as 18 bp.

Reference

1. Shimamoto, N., Toda, M., Nara, S., Komatsuzaki, T., Kamagata, K., Kinebuchi, T., & Tomizawa, J-I. Dependence of DNA length on binding affinity between TrpR and *trpO* of DNA Sci. Rep-UK, 10, 15624 (2020).
